# CURRENT STATUS OF THE MULTIDISCIPLINARY TREATMENT OF GASTRIC ADENOCARCINOMA

**DOI:** 10.1590/0102-672020180001e1373

**Published:** 2018-07-02

**Authors:** Marcelo Garcia TONETO, Luciana VIOLA

**Affiliations:** 1 Escola de Medicina, Pontifícia Universidade Católica do Rio Grande do Sul - PUCRS, Porto Alegre, RS, Brazil

**Keywords:** Stomach neoplasms, Treatment, Chemotherapy, Radiotherapy., Câncer de estômago, Tratamento, Quimioterapia, Radioterapia

## Abstract

***Background*:**

The complexity of the management of gastric cancer requires a multidisciplinary evaluation of patients with this tumor. Several treatments have been employed, associated to the surgical resection.

***Objective*:**

To review the available therapeutic alternatives for the treatment of gastric adenocarcinoma.

*****Methods*** ****:**

A review of selected articles on multidisciplinary treatment of gastric adenocarcinoma in the Pubmed and Medline databases between 2000 and 2017 was carried out. The following headings were related: stomach cancer, treatment, chemotherapy and radiotherapy.

*****Results*** ****:**

There are several valid alternatives, with good results for the treatment of gastric cancer: chemoradiotherapy or chemotherapy in the adjuvant scenario; perioperative chemotherapy; and chemoradiotherapy after neoadjuvance with isolated chemotherapy.

*****Conclusion*** ****:**

Current evidences suggest that combined multidisciplinary treatment is superior to surgery alone. However, the optimal treatment regimen is not yet established, and depends on a number of factors, especially the type of surgical resection employed. Therefore, the therapeutic decision should be made by a multidisciplinary team, assessing patient’s personal characteristics, biology of the tumor, residual disease, risks and side effects.

## RESUMO

*****Introdução*** ****:**

A complexidade do manejo do câncer gástrico torna necessária a avaliação multidisciplinar dos pacientes com esse tumor. Diversas alternativas de tratamento têm sido empregadas, associadas com a ressecção cirúrgica.

***Objetivo*:**

Analisar as alternativas disponíveis para o tratamento do adenocarcinoma gástrico.

*****Método*** ****:**

Foi realizada revisão de artigos selecionados sobre tratamento multidisciplinar do adenocarcinoma gástrico nas bases de dados Pubmed e Medline entre 2000 e 2017. Os seguintes descritores foram relacionados: câncer de estômago, tratamento, quimioterapia e radioterapia.

*****Resultados*** ****:**

Existem várias alternativas válidas, com bons resultados para o tratamento do câncer gástrico: adjuvância com químio e radioterapia ou quimioterapia isolada; quimioterapia perioperatória; e químio e radioterapia após neoadjuvância com quimioterapia isolada.

*****Conclusão*** ****:**

As evidências sugerem que o tratamento combinado é superior ao da cirurgia isolada. Entretanto, o esquema ideal de tratamento ainda não está estabelecido e depende de uma série de fatores, principalmente o tipo de ressecção cirúrgica empregada. Portanto, a decisão terapêutica deve ser tomada por equipe multidisciplinar, avaliando características pessoais do paciente, biologia do tumor, possibilidade de doença residual, riscos e a capacidade do paciente de tolerar tratamentos não isentos de efeitos colaterais.

## INTRODUCTION

Approximately 20.000 new cases of gastric cancer were diagnosed in Brazil in 2016^10^. Due to the aggressiveness of this disease, less than 20% of these patients are expected to be alive in five years, confirming that this disease remains a public health problem in the country^16^. Complete surgical resection remains as the only therapeutic alternative capable of providing a chance of cure for patients diagnosed with gastric adenocarcinoma^25^. However, at the time of the diagnosis most patients already present an advanced state of the disease, providing very low survival rates in western world. With the exception of the few patients diagnosed with early tumors, where the results of isolated surgical or endoscopic treatment are encouraging^17^, the association of other treatment alternatives, with the intention of eradicating potential micrometastases, has the objective of improving the prognosis of this disease. Notwithstanding, conflicts remain about the most adequate time to institute treatment, which chemotherapy drugs to be chosen, the use of radiotherapy, and the patients’ ideal selection^30^. 

Current management of stomach cancer has evolved in the last 20 years and the involvement of a multidisciplinary team is needed for its treatment. The alternatives that are traditionally defined by the surgeon, such as the extent of surgical resection and the lymphadenectomy, have an impact on the options of complementary treatment offered by the oncologist, making the connection between these experts essential to choose the best therapeutic options, individualizing the needs of the patients. 

The aim of this review is to study the current alternatives of complementary treatment used in gastric adenocarcinoma based on the analysis of evidence-based papers.

## METHODS

A review of relevant papers based on Pubmed and Medline databases, has been carried out between 2000 and 2017, correlating the descriptors: stomach cancer, treatment, chemotherapy and radiotherapy. From the selected articles, the following filters were applied: prospective randomized trial, humans and English language. 

## RESULTS

### Multidisciplinary treatment background

Long term survival in patients with gastric cancer has shown a gradual evolution in recent decades due to advances in surgical technique and the reduction of postoperative complications. Improvement in anesthetic techniques and in intensive care units provided the opportunity for surgeons to increase, with safety, the radicality of the procedure. Recently, there has been progress in the tumor recurrence pattern knowledge, which still occurs in more than half of the patients^5^. The understanding of this process reinforces the concept of gastric cancer as a local presentation of a systemic condition that already presents metastatic microscopic disease at the time of diagnosis. Attempts have been initiated to complement the surgical treatment with chemo and/or radiotherapy to decrease the high number of tumor recurrences. 

Despite the failure in past decades, a combined treatment effectiveness evidence began to emerge. The continuous evolution of chemotherapeutic agents associated with advances in the understanding of the tumor biology, may provide a further progress in the therapeutic alternatives, improving the effectiveness and reducing the toxicity associated with the treatment. There are currently enough evidences to indicate chemotherapy in virtually all scenarios of advanced gastric cancer. Apparently only early tumors, with no evidence of lymph node metastases, could be treated with surgical resection alone. 

It is crucial that careful staging must be performed before the therapeutic decision is established^27^ since surgery with curative intention should not be offered in the presence of metastatic disease^6^.

The most relevant papers that evaluated the gastric adenocarcinoma multidisciplinary treatment will be reviewed through a critical analysis.

### Adjuvance with chemotherapy and radiotherapy

The low survival of gastric adenocarcinoma due to high rates of local recurrence after surgical treatment were initially treated only with radiotherapy, but the results were discouraging^7^. In spite of the theoretical potential benefit, isolated chemotherapy was not considered to be beneficial as a gastric cancer adjuvant treatment^11^ in operated patients^8^. So far, stomach carcinoma was considered an unresponsive tumor to chemotherapy. The first study (INT0116/SWOG9008)^18^ that showed the benefit of combined treatment was published in 2001. It compared the results in patients’ stage IB to IV, without distant metastases, of the chemotherapy (fluoracil and leucovorin) associated with radiotherapy (45 Gy) adjuvant to surgical resection with the isolated surgery results. The average survival in the isolated surgery group was 27 vs. 36 months in the multidisciplinary treatment group (p=0.005). The analysis of the disease-free survival (31% vs. 48%) and the overall survival (41% vs. 50%), in three years, clearly favored the patients with adjuvant treatment. Relapse location analysis showed that the number of local recurrences was lower in the group undergoing treatment with chemotherapy when compared to the group of surgery alone (19% vs. 29%). The controversy of this study is that, despite the protocol recommending a D2 lymphadenectomy, 90% of the patients were operated on with an inadequate surgery for today’s patterns, with a D0 lymphadenectomy performed in approximately half of them. Although in the subgroup analysis the extent of lymphadenectomy has not shown correlation with survival, probably due to the small number of patients with D2 lymphadenectomy (n=54), further detailed analyses showed that inappropriate surgery may have adversely affected it^9^. Also, this chemoradiotherapy study group when compared to a similar group of patients operated on with D2 lymphadenectomy in Japan, the 5-year survival rate shown in the Japanese study was significantly higher^23^ suggesting that a well-performed lymphadenectomy can produce the same results as the adjuvant treatment. Another disadvantage of this treatment method is the non-negligible toxicity of the chemotherapy and radiotherapy association in the postoperative gastrectomy period^13^. Thirty-six percent of patients in the treated group were unable to complete the protocol treatment due to toxicity. 

Despite the inadequate lymphadenectomy controversy, survival benefits were statistically significant and confirmed after a 10-year follow-up analysis^26^. Therefore, the controversy whether loco-regional control can be improved with chemoradiotherapy in patients with a well-performed lymph node resection has not been clarified in this study. However, it seems clear that there is significant benefit of additional treatment with adjuvant chemoradiation therapy in patients that are operated without adequate lymphadenectomy.

### Perioperative chemotherapy

The possibility of sparing the radiotherapy side effects after an extensive surgical procedure made the idea of preoperative chemotherapy attractive. The theoretical advantages could be listed: 1) better drug release in tumor and surrounding tissues through an intact blood and lymphatic circulation; 2) downsizing of the tumor, increasing the complete resection rate and the possibility of turning unresectable tumors in resectable ones; 3) micrometastases early treatment, preventing the appearance of chemoresistant clones; 4) reduction in peritoneal cavity contamination, through a “tumor sterilization”; 5) “in vivo” therapeutic trial of the drug, allowing adjustment of the postoperative treatment according to individual response. Chemotherapy response absence, delayed surgery and increased surgical complications rates due to toxicity should be remembered as inconvenient factors for neoadjuvant treatment.

The first successful study assessing perioperative chemotherapy was known as the MAGIC trial, in which patients were randomized to surgical treatment alone or to perioperative chemotherapy. Seventy-four percent of the tumors were located in the stomach, 14% in the distal third of the esophagus, and 11% classified as of esophageal-gastric transition tumors. Tumor stage should be T2 or higher. Chemotherapy consisted of three preoperative cycles with the use of epirubicin, cisplatin and intravenous 5-fluoracil, followed by surgery and, afterwards, three more postoperative cycles with the same medications. The fear of an increase in postoperative complications was not confirmed since the morbidity (46% vs. 45%) and mortality (5.6% vs. 5, 9%) were similar in both groups. In pathological evaluation of the specimens, there was a decrease in tumor size (3 cm vs. 5 cm, p<0.001), a higher proportion of T1 and T2 tumors (51.7% vs. 36.8%, p=0.002) and N0 and N1 (84.4% vs. 70.5%, p=0.01) in the treated group, consistent with a tumor reduction caused by chemotherapy. The five-year survival was of 36% in the chemotherapy group and of 23% in the isolated surgery one. This study demonstrated that, despite the absence of radiotherapy, there was a clinically significant reduction in tumor recurrence and mortality, presenting as a viable alternative for the treatment of gastric cancer. It should be noted that of the 250 patients enrolled in the chemotherapy group, 215 (86%) completed the three preoperative chemotherapy cycles and only 104 (41.6%) of them were able to complete the three postoperative chemotherapy cycles. Neutropenia was the most significant toxicity occurring in 23% of the individuals. Less than 12% of them have presented severe toxicity. Due to the inconvenience of the continuous fluorouracil infusion, an epirubicin, oxaliplatin, and capecitabine scheme was compared to ECF in a prospective randomized study for patients with metastatic disease, demonstrating that the overall survival rate was similar in both groups (11.2 vs. 9.9 months)^4^. Despite the promising results, the main limitations of this study again concern the extension of lymphadenectomy, which was inadequate in about 40% of the patients, the inclusion of a large number of tumors of the distal esophagus and gastroesophageal transition, and the high percentage of patients who failed to complete the initially planned chemotherapy. The absence of a standardized preoperative staging is also criticized in this study.

A recent phase III randomized study, conducted in 28 centers in France has also shown similar positive results with 5-year survival rates favorable to the perioperative chemotherapy group (38% vs. 24%)^29^. Importantly, most patients in this study had tumors located in the distal esophagus or gastroesophageal junction (75%). 

In summary, perioperative chemotherapy superiority when compared to isolated surgery has been confirmed in solid studies and with a well-designed methodology. There are no differences in length of stay, rates of complications and operative mortality, and there seems to be an improvement in the histopathological tumor evaluation^15^. Among the many perioperative chemotherapy studies in progress, the results of the FLOT4 trial, which confronts the ECF schemes using docetaxel with 5-Fu and oxaliplatin are pending. Preliminary results showed a 16% complete pathological tumor response rate, with acceptable tolerability of adverse reactions^1^. A high expectation about survival results is generated on this study due to the good patient treatment tolerability.

### Combined treatments in patients with D2 lymphadnectomy

Significant differences are detected in patients’ survival treated for gastric adenocarcinoma when comparing treatment in Japan to those in the West. Despite the hypotheses of differences in tumor biology, there is no evidence to support this claim^19^. Untill the early 2000s, the standard treatment for gastric adenocarcinoma in Japan was based only on gastrectomy with D2 lymphadenectomy, and the results were far superior to any combined treatment in the rest of the world. An adjuvant chemotherapy study^22^ was started after phase II studies have shown response rates greater than 40%, with the oral-ingestion S-1 drug, derived from 5-fluoracil. The proposition was to measure the use of chemotherapy, started six weeks after surgery and administered by a year. After enrollment and randomization of 1509 individuals in stages II and III, the first interin analysis demonstrated significantly better results in patients treated with chemotherapy (three-year survival rate: 80% vs. 70%, p=0.003), and the study had to be discontinued. Further data analysis has confirmed a 33% increase in 5-year survival for patients receiving chemotherapy, with an overall survival of 71.7% vs. 61.1%^24^. Surgical procedure quality is highlighted in this study, since more than 99.8% of the patients underwent adequate lymphadenectomy. 

These data support the current guidelines in Japan, recommending adjuvant chemotherapy as a standard treatment for patients stage II-III, after a R0 resection with D2 lymphadenectomy^12^. Unfortunately, the drug used in this study demonstrated a different kinetic behavior in caucasians, affecting the drug tolerance and efficacy in the Western population. 

Another recent study was the CLASSIC^2^, conducted in Asia, in which the XELOX scheme was applied for eight cycles in 1035 individuals after gastrectomy with D2 lymphadenectomy for locally advanced tumors. After a mean follow-up of 34.4 months, disease-free survival for the treated group was significantly higher in three years (74% vs. 60%; p<0.0001). Grade 3 or 4 adverse events occurred in approximately half of patients with severe toxicity reported in 7% of them, consistent with the known safety profiles of the chemotherapy treatment. The study data definitive publication showed that the chemotherapy group 5-year survival was 78% against 69% of those that underwent surgical resection alone, with a 31% reduction in tumor-related risk of dying^20^.

Based on the results of these studies, it is strongly suggested that adjuvant treatment after resections with D2 lymphadenectomy bring clinical benefits to patients with resectable gastric adenocarcinoma. The argument that an excellent surgery cancels the benefits of chemotherapy virtually ends with these results. Meta-analysis with a significant number of patients (3838) demonstrated a 5.8% absolute advantage in the 5-year survival (49.6 vs. 55.3%) and 7.4% in 10 years (37.5 vs. 44.9%) in patients treated with adjuvant chemotherapy following gastrectomy^21^.

### Does the radiotherapy association increase the benefit of patients submitted to D2 lymphadenectomy?

The ARTIST^14^ study was conducted to compare the outcome of adjuvant chemotherapy alone with chemotherapy associated with radiotherapy in patients undergoing gastrectomy with D2 lymphadenectomy. Four hundred and fifty-eight patients submitted to D2 lymphadenectomy with complete tumor resection were included. The group submitted to chemotherapy alone received six cycles of cisplatin and capecitabine. The other arm received two cycles of the same medication followed by chemoradiotherapy (45 Gy radiation, associated with capecitabine 825 mg/m^2^ twice daily) and two more cycles of chemotherapy. Disease-free survival in both groups was equivalent, questioning the radiotherapy benefit in patients D2 resection. The authors conducted a subgroup analysis in patients with positive lymph nodes. This subgroup showed a significant increase in disease-free survival patients undergoing chemoradiotherapy (77.5% vs. 72.3%) in a 3-year period. This study controversy was due to the fact that approximately 60% of the tumors were stages II and IIIA, therefore with a better prognosis. This trial another important contribution is the new prospective randomized study design (ARTIST-II) comparing the results only in patients with positive lymph nodes.

It is under initial assessment, in Europe, the CRITICS^28^ study, which compares three chemotherapy cycles with epirubicin, cisplatin and capecitabine followed by surgery. After resection, patients are randomized to receive three cycles of such drugs or 45 Gy of radiotherapy in combination with capecitabine and cisplatin. The assessment included patients in stages IB to IVA, based on endoscopic ultrasound and computed tomography. Only 17% of the tumors were from gastroesophageal junction. D3 lymphadenectomy was performed in 37% of the patients. There was no difference either in the patient’s survival with or without radiotherapy or in a 5-year time surviving rate.

These data analysis, originating from well-conducted randomized prospective trial, suggests that postoperative radiation therapy does not add survival improvements to patients with negative lymph nodes, when surgical resection has been well performed. ARTIST-II results are being expected for the definition of its potential benefit in patients with lymph node involvement, and its use should currently be restricted to patients that are submitted to inadequate lymphadenectomy.

## CONCLUSION

Locally advanced gastric adenocarcinoma still remains a difficult disease to be treated. Tumor resection with free margins and adequate lymphadenectomy remains as the greatest chance of cure for patients with this aggressive neoplasm. However, with the exception of patients with early gastric tumors with low risk of lymph node metastases, evidences show that increase in survival rates is not possible with only one treatment modality. Chemotherapy and radiotherapy association increases survival in patients undergoing surgery, especially in those that were not exposed to an adequate lymph node dissection. Perioperative chemotherapy does not increase surgical complication rates, it may increase the proportion of resected patients and the overall survival. Isolated chemotherapy improves the survival of patients that underwent D2 lymphadenectomy. Recent researches have shown treatment improval with effective chemotherapeutic and better tolerability agents, and also with the radiotherapy enhancement systems. It is expected for the near future that a better understanding of stomach cancer molecular bases is found, which allows the incorporation of new therapeutic targets. Promising advances in immunotherapy already shown in other tumors should be introduced and could add benefits in this disease future treatment. The treatment decision should be taken by a multidisciplinary team, assessing the patient’s personal characteristics, the tumor aggressiveness, residual disease, surgical risks and the patient’s ability to tolerate treatments that are not free of side effects. Technological advances and the development of new therapeutic alternatives indicate that the future of gastric cancer treatment is promising. However, it still depends on an appropriate and safe surgical procedure.
